# The Treatment of Acne Rosacea

**Published:** 1855-06

**Authors:** James Morris


The Treatment of Acne Rosacea. By James Morris, M. D., F. R. C. S.—Acne Rosacea has, perhaps, received a sufficient share of attention to necessitate some apology for writing on the subject. My apology must be sought in the recommendations which I have to give with respect to its treatment. Those who dip into the literature of this disease will have no reason to complain of the paucity of remedies recommended, and the copious list is generally preceded by some discouraging presage, under the head of prognosis; thus Rayer says, "Cuperosa is curable when the subject of it is young, the eruption recent and slight, and the pustules not very numerous. On the contrary, when it shows itself in adult age, is connected with chronic affection of the digestive organs, or when it is hereditary, of long standing, and of large extent, the best treatment rarely succeeds." Schedel calls it "an obstinate disorder, and in many cases incurable." Erasmus Wilson finds it "very intractable;" and Dr. Copland considers the "treatment generally unpromising."

It would seem that in all this the disease is but supporting an ancient reputation, for Falstaff is made to say to Bardolph—"Do thou amend thy face, and I'll amend my life." As considerable confusion has arisen in the works of dermatologists, by unnecessarily increasing the number of species of acne, in order to remove any doubt to which form of the disease I am referring, I quote a description by Erasmus Wilson, from his popular work on the Skin, the comic vein of which must be pardoned on account of the accuracy of the picture. After proposing for the complaint the new synonym of the "besieger," he says, "It begins, for example by very slowly throwing up a mound of red skin, and for months, perhaps, goes no further; then we see a chain of low barrows, extending to another mound in the act of rising; sometimes two chains passing in different directions, or perhaps a mound in the distance, which the chain goes to meet. These operations are always remarkable for their slowness, but not less for their certainty of success, for wherever the skin is hemmed in by these pimply ridges, it is as deeply red as the pimples themselves; and after a time, when the whole nose, probably, and a considerable extent of the adjacent face is covered by them, both the latter and the intermediate skin are marked by large veins, which spread out their branches like tributary streams of a large river. Occasionally, small collections of yellow matter form upon these pimples, but this is by no means their common character. They preserve a heightened color under all circumstances, but undergo a change having relation to the state of the blood flowing through them. Thus, for example, when the circulation is active, they are fully distended and brightly red; while, in cold weather, or under the influence of causes which retard circulation, they are bluish or even livid in hue."

In discussing the appropriate treatment of this affection, one most important question meets us at the outset. It is, or is it not, generally symptomatic of disease of the liver? And this question resolves itself into two, which require separate consideration. First, does it frequently accompany serious disease of the liver? To this I think it must be answered that the association is not rare. The second question is, what relation does it bear to the hepatic affection? To this I would reply merely that of accidental co-existence. This distinction has not been adequately attended to. That the association with disease of the liver cannot be very infrequent is to be inferred partly from the impossibility of otherwise accounting for the general belief that the two maladies are in some way related; and, besides that, I have myself seen two unmistakeable instances of this connexion. I find Dr. Copland saying, "A rosacea often precedes serious disease of the liver more frequently co-exists with it, and most commonly indicates a congested and obstructed state of this viscus." It is needless to multiply quotations, but many such are to be found. This doctrine appears to have controlled the treatment in a most mischievous manner, and it has done so the more effectually that, in as far as co-existence is concerned, it is founded in fact. Thus, the author above quoted recommends blood-letting, leeches to the liver, thighs, and feet, liquor potassæ, with many other things, but he makes no mention of tonics. Ambrose Paré went further; he advised copious blood-letting—a proceeding doubtless capable of blanching the reddened parts; besides taking blood from the basilic vein, the forehead, and the nose, he recommends leeches over the face, and cupping between the shoulders; but all this may perhaps have been moderate in his time. That the suppression of this eruption is not followed by any injurious effect upon the liver, is inferred on these grounds; besides curing a considerable number of cases where the liver was unaffected, the patients, with scarcely an exception, enjoyed afterwards better health than they had done before. I have also cured the eruption, with only the simplest precautions, in two cases of hepatic affection, the occurrence of jaundice, with induration and enlargement of the liver, together with other symptoms, removing all doubt from the diagnosis; and certainly no unfavorable effect was manifested upon the disease, the patients having remained long afterwards under my observation. Moreover, acne rosacea is now recognised as a disease of the subaceous follicles; it is indeed probable that the fatty secretion of these follicles may to some extent relieve the liver; but it may be fairly asked which is likely to give most relief, an inflamed or a healthy gland? We know that in glands the first sign of subsiding inflammation is generally restoration of the natural secretion, and the effect of the treatment proposed for this affection is not to suppress the subaceous secretion, but to restore it to a healthy state. Rayer, who connects his cuperosa with chronic gastro-enteritis, adds—"Its dependence on an affection of the liver is more rare and difficult to ascertain, notwithstanding the old and repeated assertions to the contrary." Plumbe disbelieved even the association with disease of the liver, though he says, "In the only case I have met with, where hard drinking did not form part of the habits of the patient, an extraordinary appetite, not of the most delicate kind, was well known to exist." This last, I think, is not borne out by later experience. He recommends, however, a "tonic plan," which, he says, he "had followed with pretty uniformly successful results." My own cases lend strong support to this recommendation of Plumbe; and though it would be rash to affirm that such cases require depletive treatment, yet they must be in very small proportion to those requiring tonics. Now, purgatives are useful adjuncts in females; where the disease has been accompanied by leucorrhœa, a frequent occurrence, striking benefit has been derived from the use of iron mixture with compound decoction of aloes. In several instances palpitation, and, in one, morbus cordis, from previous rheumatism, were associated with the eruption. The local treatment is of the highest importance, and the agent which I wish to place more prominently before the profession for this purpose is sulphur. I advocate its claims with the more freedom, as it is no new remedy, and has obtained weighty suffrages in past years. Thus Rayer says—"Cold sulphurous applications, *en douche* and *en arrasoir*, are very efficacious in restoring the skin to its natural state." Other similar opinions might be added. In one remarkable instance this application readily effected a cure after the patient, a lady, had suffered the vexation of the complaint for twenty years, and had undergone an immense variety of treatment without benefit, ending with the homœopathic; in other cases the same result speedily followed after four and six years. It would seem that the sulphur works its way into the sebaceous follicles, where it probably dissolves to some extent in the oily secretion. It has been recommended as an internal remedy, in which case it might reach the same position, as it is eliminated by these glands when taken for any period. The form in which I have invariably used it, is recommended by Mr. Erasmus Wilson for the treatment of acne in geueral. In my hands it has appeared to possess little, if any, infiuence over the punctated form; while for the rosaceous variety it appears to be the remedy. It is as follows:—A drachm of camphor is pulverized with alcohol; twice as much milk of sulphur is then added, (Mr. Wilson recommends sulphur sublimatum;) afterwards distilled water, to render it sufficiently liquid for use. This lotion is smeared with the finger over the face freely at night, and more sparingly in the morning; the effect is generally very soon apparent, and is often most striking. The lac sulphuris often contains as much as sixty per cent., of sulphate of lime; this does not appear to interfere with the action of the remedy. This preparation is preferable, on account of its minute sub-division, which makes it less irritating, and also for its whiteness. The use of this lotion should be persisted in for a considerable period if the blotches return; and if the surface be thus preserved in a healthy state, Nature will gradually restore the deeper-seated structures to their normal condition. It does not appear to lose its efficacy by continued use. In conclusion, this annoying malady cannot be concealed; it obtains little commiseration; and if it be rendered in any degree a rare spectacle by the general adoption of what the writer believes to be an improved treatment, he will have amply attained his object in writing this paper.—*Lancet.*

